# When genomes burn: how DNA damage ignites inflammation and fuels ageing

**DOI:** 10.1098/rstb.2025.0337

**Published:** 2026-11-12

**Authors:** Emmanouil Theodorakis, George A. Garinis

**Affiliations:** ^1^ Department of Biology, University of Crete, Heraklion, Greece; 2 Institute of Molecular Biology and Biotechnology, Foundation for Research and Technology-Hellas, Heraklion, Greece

**Keywords:** genome instability, DNA damage response, chronic inflammation, cellular senescence, ageing

## Abstract

Ageing is accompanied by a progressive loss of homeostasis driven by inter connected molecular stress responses. Two central hallmarks, genome instability and chronic low-grade inflammation (inflammaging), are increasingly recognized as mechanistically inseparable. Persistent DNA damage, defective repair and transcriptional stress activate inflammatory signalling not only through canonical DNA damage response (DDR) pathways, but also via innate immune sensing of mislocalized self-nucleic acids. In turn, inflammatory mediators, metabolic stress and reactive oxygen and nitrogen species inflict new genomic damage and compromise repair, establishing a self-reinforcing feedback loop. Evidence from human DNA repair syndromes and genetically engineered mouse models reveals that chronic DDR engagement rewires metabolism, endocrine signalling, mitochondrial function and immune surveillance well before overt tissue degeneration. Recent work further highlights extra-nuclear DDR programmes, RNA-based danger signals and organelle dysfunction as key amplifiers linking genome instability to inflammation. Here, we synthesize insights from genome maintenance, innate immunity and metabolism to propose a unified framework in which ageing emerges from the chronic persistence of stress circuits originally evolved for transient repair and host defence. We discuss evolutionary implications, disease relevance and emerging therapeutic strategies aimed at disrupting this damage–inflammation axis to restore tissue homeostasis.

This article is part of the theme issue ‘Developmental circuits in ageing’.

## 1. The intertwined hallmarks of ageing

Ageing reflects a progressive loss of physiological integrity as damage-control systems fail over time. Among the established hallmarks of ageing, DNA damage, genome instability and chronic low-grade inflammation (inflammaging) occupy a central position [1–3]. DNA damage comprises chemical or structural alterations that disrupt the normal integrity of DNA, including abasic sites, bulky adducts, oxidative lesions, DNA-protein crosslinks, interstrand or intrastrand crosslinks and single- or double-strand breaks (SSBs and DSBs). Unlike DNA damage, mutations are permanent, heritable changes in DNA sequence or chromosome structure, such as base substitutions, insertions, deletions, copy-number alterations and chromosomal rearrang-ements. Genome instability describes the increased tendency of cells or tissues to accumulate such mutations and chromosomal abnormalities over time. DNA damage is therefore a major driver of genome instability, but it is not synonymous with mutation, because DNA lesions may impair replication or transcription without necessarily becoming fixed as sequence changes. Although genome maintenance and immune dysregulation have often been treated as distinct hallmarks, current evidence shows they are deeply interdependent and self-reinforcing [4–6]. Persistent DNA lesions and defective repair elicit chronic stress responses that activate inflammatory signalling, while inflammatory mediators and metabolic by-products further erode genome integrity. This reciprocal relationship establishes a feed-forward circuit that accelerates tissue dysfunction and organismal ageing. Work on human DNA repair-deficient progeroid syndromes and their mouse counterparts has been instrumental in exposing this axis [7–9]. In models deficient in nucleotide excision repair (NER), transcription-coupled repair (TCR) or ERCC1-XPF-dependent crosslink repair, unrepaired DNA lesions have effects that extend beyond their local accumulation. NER removes bulky, helix-distorting lesions, whereas TCR selectively eliminates transcription-blocking lesions from actively transcribed genes. The ERCC1-XPF endonuclease functions in NER, as well as in TCR, and also contributes to the processing of DNA interstrand crosslinks and other complex DNA repair intermediates. The critical role of TCR in maintaining tissue homeostasis is underscored by TCR disorders such as Cockayne syndrome, where defective removal of transcription-blocking DNA lesions results in severe premature ageing phenotypes, particularly affecting post-mitotic organs and tissues. Failure of these pathways allows transcription-blocking lesions to persist, leading to broad transcriptional reprogramming, endocrine alterations and systemic inflammatory responses that recapitulate features of natural ageing [10–13]. These include suppression of the somatotroph axis [14–17], chronic autoinflammation [18] and tissue-specific degenerative phenotypes such as neurodegeneration [19], lipodystrophy [18,20] and organ fibrosis [18]. Studies of transcription-blocking lesions and replication stress indicate that DNA damage drives organismal decline through defined and regulated signalling pathways rather than through passive wear and tear alone. Persistent lesions disrupt transcription, chromatin organization and cellular secretory programmes, thereby linking genome instability to systemic dysfunction. A central question is how DNA damage promotes inflammation and, conversely, how inflammatory signalling reinforces genomic instability. This reciprocal interaction appears to converge on shared nodes connecting the DNA damage response, innate immune pathways and metabolic stress sensors. Although transient activation of these systems supports repair and host defence, their chronic engagement establishes a maladaptive state of sustained cellular alarm that perpetuates both damage and dysfunction. We therefore propose that ageing and age-related disease are driven, at least in part, by a self-amplifying damage–inflammation axis in which unrepaired DNA damage activates inflammatory signalling, which in turn further compromises genome stability.

## 2. DNA damage as an inflammatory trigger

The DNA damage response (DDR) orchestrates a conserved surveillance network that detects, signals and repairs genomic lesions to preserve cellular and organismal integrity [10,21] (figure 1(1)). Central to this system are the phosphatidylinositol 3-kinase-related kinase (PIKK) family kinases ataxia-telangiectasia mutated (ATM) and ataxia telangiectasia and Rad3-related (ATR). ATM is primarily activated by DNA double-strand breaks through the MRN (MRE11-RAD50-NBS1) complex, whereas ATR is activated by replication stress and regions of replication protein A (RPA)-coated single-stranded DNA (ssDNA), such as those arising at stalled or collapsed replication forks [22]. Importantly, ATM can also be engaged by transcription stress, as transcription-blocking lesions, spliceosome displacement, ssDNA intermediates and R-loops can initiate non-canonical ATM signalling [23]. Upon activation, ATR and ATM preferentially signal through checkpoint kinase 1 and 2 (CHK1 and CHK2), respectively, and converge on downstream effectors including p53, enforcing cell-cycle checkpoints, coordinating DNA repair pathway choice and limiting the propagation of genome instability (figure 1(2)). In its acute form, the DDR is protective and self-limiting. With age, or under persistent genotoxic stress, this response can become chronically engaged. Sustained ATM/p53 activity establishes persistent checkpoint signalling and drives transcriptional reprogramming towards inflammatory and secretory states, typified by cellular senescence and the senescence-associated secretory phenotype (SASP) [24,25].

**Figure 1.**
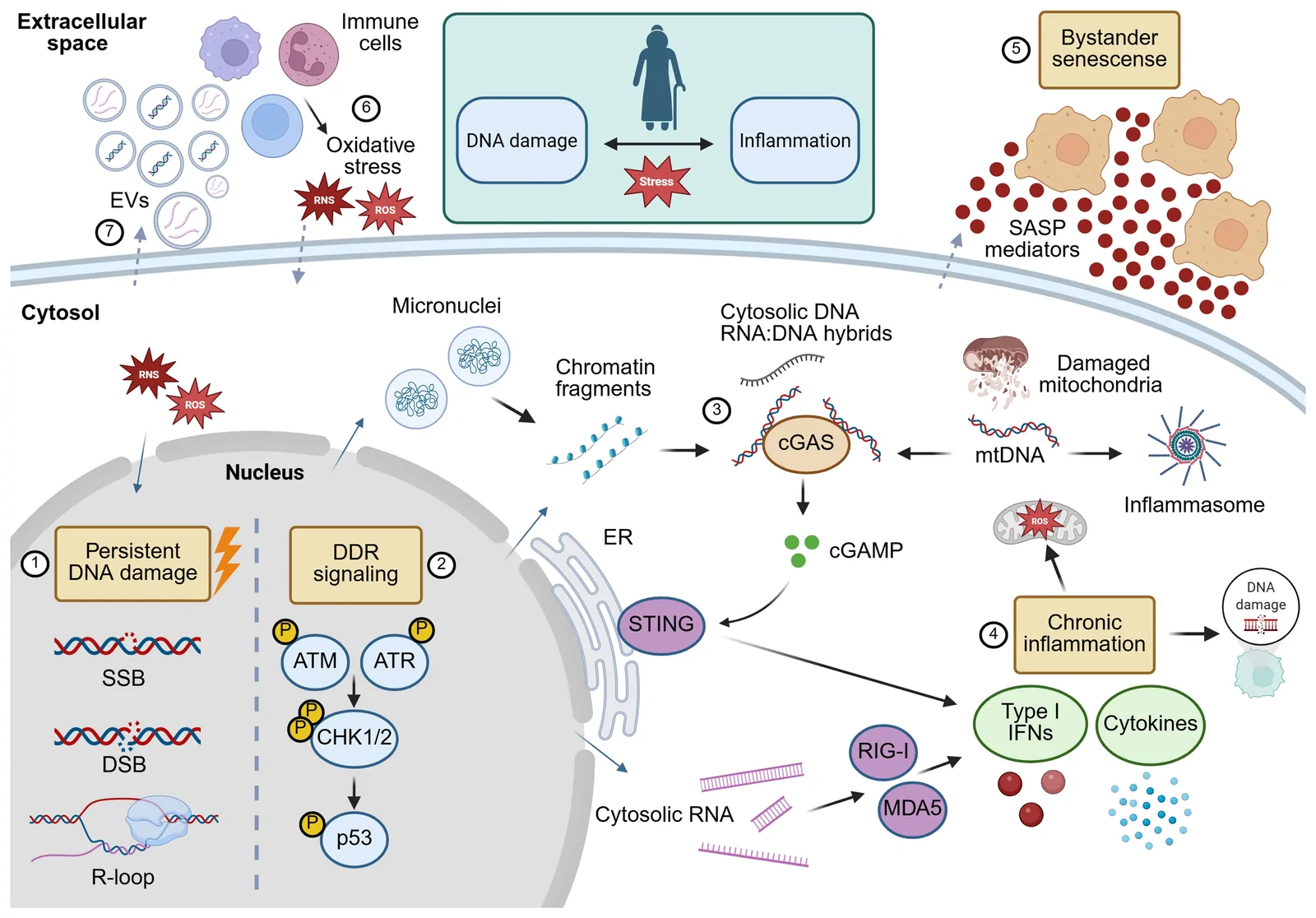
The damage–inflammation axis linking genome instability to chronic inflammatory signalling. Persistent DNA damage induces sustained DNA damage response (DDR) signalling (1,2). Chronic DDR promotes genomic instability and the generation of aberrant nucleic-acid species. Nuclear lesions, including strand breaks and R-loop-associated structures, drive micronuclei formation and release of chromatin fragments into the cytosol. Cytosolic DNA, RNA:DNA hybrids and mitochondrial DNA derived from damaged mitochondria activate innate immune pathways, most prominently the cGAS-STING axis, leading to cGAMP production and induction of type I interferons and pro-inflammatory cytokines (3). Cytosolic RNA species activate RIG-I-like receptors, further amplifying inflammatory signalling. Mitochondrial dysfunction additionally promotes inflammasome activation. The resulting inflammatory milieu establishes chronic inflammation, which feeds back on genome integrity through oxidative and nitrosative stress, impaired DNA repair and replication stress (4). Senescent cells exacerbate this process through secretion of senescence-associated secretory phenotype (SASP) factors, promoting bystander senescence (5). Immune cells and extracellular vesicles propagate inflammatory and genotoxic signals across tissues and organs (6,7). Together, these interconnected processes form a self-sustaining damage–inflammation axis that drives inflammaging and age-associated diseases. (Created in BioRender: Theodorakis E. (2026) https://BioRender.com/lix727r.)

In NER- and ERCC1-XPF-deficient progeroid mice, persistent transcription-blocking lesions and DNA interstrand crosslinks trigger chronic ATM-dependent signalling and derepression of pro-inflammatory cytokines in metabolically active tissues, including adipose tissue and liver [18,26]. We have shown that these secretory changes are cell-autonomous in adipocytes and arise before overt tissue degeneration. They are sufficient to recruit immune cells, induce lipodystrophy and establish chronic tissue inflammation. Because DNA repair is compromised, DNA lesions persist, and damaged cells remain locked in a chronic stress-signalling state, continuously producing pro-inflammatory cytokines, growth factors and proteases that remodel the tissue microenvironment and propagate stress to neighbouring cells. In parallel, genome instability promotes the leakage of nuclear and mitochondrial DNA into the cytoplasm, where it activates nucleic acid-sensing pathways such as cGAS-STING [27–29] (figure 1(3)). Originally evolved as part of antiviral defence, this pathway amplifies DDR-derived signals through type I interferons and nuclear factor κB (NF-κB)-dependent cytokines, thereby linking persistent DNA damage directly to immune activation. Studies in DNA repair-deficient and ageing models have shown that cytosolic DNA, RNA:DNA hybrids and transcription-stress products accumulate in tissues with high metabolic or transcriptional demands, including pancreas, brain and liver [9,13,19,30,31]. As a result, the DDR shifts from a cell-autonomous repair mechanism to a paracrine danger-signalling system that promotes chronic inflammation (figure 1(4)). While beneficial when transient, this conversion from repair to secretion becomes detrimental when sustained, initiating a feed-forward cycle of inflammaging and tissue degeneration.

A central conduit linking the DDR to inflammation is the generation and mislocalization of self-derived nucleic acids. Under normal conditions, genomic DNA is confined to the nucleus and mitochondria, preserving innate immune tolerance. When this compartmentalization fails—through replication stress-induced chromatin bridges, micronuclei formation followed by nuclear envelope rupture or mitochondrial DNA release during oxidative injury or defective mitophagy—self-DNA gains access to the cytosol and is perceived as a danger signal. In this context, cGAS detects cytosolic DNA and produces cyclic GMP-AMP (cGAMP), which activates STING at the endoplasmic reticulum and induces type I interferon and NF-κB-dependent inflammatory programmes. Persistent activation by self-DNA promotes cellular senescence, sterile inflammation and age-related pathology. Importantly, self-DNA sensing is not determined solely by DNA abundance. It is also constrained by chromatin context, because nucleosomes can competitively inhibit DNA-dependent cGAS activation and thereby impose a threshold for aberrant inflammatory signalling [32]. Cytosolic RNA sensing further amplifies inflammation in response to genome instability. Retinoic acid-inducible gene I (RIG-I)-like receptors, including RIG-I and melanoma differentiation-associated protein 5 (MDA5), detect aberrant RNA species generated by transcriptional stress, derepressed genomic regions or defective RNA processing, thereby reinforcing inflammatory gene-expression programmes [33,34]. In parallel, transcription-blocking lesions or impaired elongation can promote the accumulation of RNA:DNA hybrids as R-loops, which may become immunogenic when improperly processed. Consistent with this model, disrupted nuclear R-loop metabolism can generate cytoplasmic RNA:DNA hybrids that engage pattern-recognition receptors, including cGAS, directly linking transcriptional stress to innate immune activation [35]. In addition to canonical immune sensors, multiple genome maintenance factors act as non-canonical regulators of immune tolerance by preventing the accumulation of immunostimulatory nucleic acids. The exonuclease three prime repair exonuclease 1 (TREX1) degrades cytosolic DNA derived from replication intermediates and endogenous retroelements [36]; its loss results in pathological STING activation and autoimmune syndromes. Similarly, the RNase H2 complex resolves RNA:DNA hybrids formed during replication and repair, and mutations in its components cause Aicardi–Goutières syndrome, a prototypical interferonopathy driven by endogenous nucleic acids [37]. Interestingly, studies have shown that RNA:DNA hybrids induced by transcription-blocking lesions can contribute to the generation of immunostimulatory ssDNA fragments in the cytosol, triggering viral-like inflammatory responses in ERCC1-deficient pancreata and in naturally aged tissues [31]. Together, these findings support a broader view in which genome instability progressively erodes nucleic-acid ‘self-tolerance’, converting by-products of damage processing into endogenous danger cues that sustain inflammaging [38].

The integrity of the nuclear envelope provides another essential layer of control in the genome–immune interface. Far from a passive barrier, the envelope maintains the physical segregation of chromatin from cytosolic sensors. Mechanical stress, defective lamin architecture or persistent DNA instability can compromise this barrier, allowing chromatin fragments to enter the cytoplasm and activate innate immune pathways [39]. Ageing and genotoxic stress progressively weaken nuclear mechanics. Mutations or post-translational modifications in lamins A/C, as seen in Hutchinson-Gilford progeria syndrome, cause transient ruptures that permit leakage of nuclear material. Micronuclei, frequent products of chromosomal mis-segregation, can undergo catastrophic envelope collapse, releasing chromatin to cytosolic sensing machinery and promoting inflammatory signalling. Persistent DDR signalling can also reshape nuclear–cytoplasmic communication by altering nuclear pore composition and transport, facilitating export of damage-associated RNAs and chromatin fragments. The appearance of nuclear material extending through a weakened nuclear lamina or sites of nuclear envelope rupture is now recognized as a hallmark of senescent and aged cells, providing a striking visual manifestation of nuclear envelope failure and the initiation of innate immune signalling [40].

Mitochondria integrate into this circuit as both targets and amplifiers of damage-associated inflammatory signalling. Persistent nuclear damage can drive mitochondrial dysfunction and mitochondrial DNA (mtDNA) release, activating both cGAS-STING and inflammasome-associated pathways [41,42]. mtDNA release is increasingly recognized as a potent innate immune trigger that can act intracellularly, extracellularly and systemically, thereby amplifying sterile inflammation during ageing [43]. The outcome is an integrated nuclear–mitochondrial inflammatory circuit in which structural collapse and metabolic stress reinforce one another to sustain low-grade inflammation. Together, these interconnected processes illustrate how persistent DNA damage transforms the genome's protective surveillance system into a chronic alarm network. Breaches in nuclear and mitochondrial integrity, coupled with innate sensing of mislocalized nucleic acids and transcriptional stress intermediates, convert the by-products of genome instability into drivers of inflammation. The resulting communication between the DDR, cytosolic sensing pathways and organellar stress responses constitutes the molecular foundation of the damage–inflammation axis, a key engine of cellular ageing and tissue degeneration. These key nodes and feedback transitions that connect DNA instability to inflammatory signalling are summarized schematically in figure 1.

## 3. Inflammation as a genotoxic stressor

While DNA damage can initiate inflammation, inflammation itself is inherently genotoxic [44]. Activated immune cells, particularly neutrophils, macrophages and microglia, release reactive oxygen and nitrogen species (ROS and RNS) as part of their antimicrobial arsenal. These reactive molecules diffuse into surrounding tissues and generate oxidative and nitrative DNA lesions, including 8-oxoguanine, abasic sites, single-strand and double-strand breaks and DNA crosslinks, thereby directly increasing genome instability. Chronic inflammation is, therefore, not only a downstream consequence of genome instability, but a persistent upstream source of endogenous genotoxic stress [45]. Neutrophil activation can directly induce oxidative DNA lesions and strand breaks in adjacent epithelial cells [46], and neutrophil-driven genomic instability can, in turn, prolong inflammatory pathology and impair tissue repair [47]. A key mechanism of inflammation-driven DNA damage is nitric oxide-dependent genotoxicity. *In vivo*, inducible nitric oxide synthase (iNOS)-dependent nitrosative stress has been directly linked to inflammation-associated DNA instability [48]. Inflammatory cytokines can further promote nitric oxide-dependent DNA damage while concomitantly inhibiting repair capacity, shifting the balance towards persistent lesions and mutagenesis [49]. Consistent with this, interleukin-22 (IL-22)-driven inflammatory signalling promotes nitric oxide-dependent DNA damage and dysplasia in colitis-associated carcinogenesis [50]. In human tissues, nitrative DNA lesions such as 8-nitroguanine serve as biomarkers linking chronic inflammation to DNA instability in inflammation-associated carcinogenesis [51]. Together, these findings support the concept that inflammation establishes a ‘genotoxic niche’ that favours the accumulation and persistence of DNA lesions [45].

Beyond direct chemical injury, inflammation signalling promotes genome instability by actively reshaping cell-cycle behaviour and replication dynamics. Pro-inflammatory mediators such as tumour necrosis factor 1α (TNFα) and interleukin-1β (IL-1β) activate NF-κB and mitogen-activated protein kinase (MAPK) pathways that stimulate proliferation and survival in stressed cells, forcing replication across unrepaired lesions and increasing replication stress [52]. This is particularly important because replication stress is not only a driver of DNA breaks, but also a potent trigger of cytosolic DNA accumulation and innate immune activation [53]. Consistent with this view, NF-κB acts as a central node that translates stress into long-term inflammatory programmes and contributes to persistent senescence-associated signalling in ageing and progeroid contexts [26]. One more layer of inflammatory genotoxicity arises from infection-associated inflammatory microenvironments. Pathogen-driven inflammation can also generate DNA strand breaks while simultaneously engaging inflammatory transcriptional programmes; for example, *Helicobacter pylori* infection induces DNA strand breaks that contribute to NF-κB-dependent gene expression [54]. *In vivo*, infection-induced colitis is accompanied by dynamic stress responses and DNA damage programmes that precede inflammation-associated tumorigenesis [55]. These examples illustrate that inflammation-linked genotoxicity is not restricted to sterile settings, but is a broad biological response to immune activation that becomes pathological when chronically sustained [56].

In addition, inflammation can increase genomic instability through transcriptional stress and aberrant nucleic-acid metabolism. Our recent work has shown that transcriptional stress, whether imposed by bulky lesions stalling RNA polymerase II (RNAPII) or by defects in transcription elongation factors such as TFIIS, leads to R-loop accumulation, telomere uncapping, chromatin bridges and the release of telomeric DNA fragments into the cytosol and extracellular vesicles [1,30]. These telomeric fragments act as potent danger signals, inducing paracrine senescence in neighbouring cells and extending inflammatory genotoxic stress beyond the initiating cell population. Such observations suggest that inflammation does not merely cause stochastic damage, but can propagate structured, damage-associated signals through extracellular communication. Chronic inflammatory signalling can further sensitize tissues to genotoxic stress by sustaining ROS/RNS exposure and engaging pathways linked to DNA damage responses and senescence. In inflammatory bowel disease, macrophage- and nitric oxide-associated programmes correlate with DDR pathway engagement and senescence-associated signatures [57]. In tissues with ongoing inflammation, such as liver, adipose tissue and brain, this paracrine genotoxic stress becomes self-reinforcing. DNA instability in resident cells induces senescence and SASP production (figure 1(5)), which recruit immune cells that release more ROS and RNS, closing a destructive feedback loop (figure 1(6)). In adipose tissue, for instance, we have shown that persistent DNA damage in ERCC1-deficient adipocytes induces ATM-dependent derepression of pro-inflammatory cytokines, recruitment of leukocytes and progressive fat depletion, establishing a causal link between DNA damage-driven autoinflammation and lipodystrophy [6,18]. In the pancreas, DNA damage-induced R-loops and ssDNA accumulation drive chronic pancreatitis and fibrosis, illustrating how inflammatory signalling and genotoxic stress converge to remodel tissue architecture [31].

Inflammation also reshapes the DDR itself, reprogramming it from a transient repair network into a chronic stress circuit. Persistent exposure to pro-inflammatory cytokines such as TNFα, interleukin-6 (IL-6) and type I interferons has been shown to sustain low-level ATM signalling and p53 phosphorylation even in the absence of overt DNA lesions, thereby maintaining a chronic or ‘pseudo-DDR’ state [54,57]. This persistent signalling promotes senescence and SASP production and can lock tissues into long-term inflammatory remodelling [47,57]. Importantly, this illustrates that inflammation is not simply damaging DNA, but also altering how damage is sensed, processed and resolved [45]. Moreover, inflammatory signalling can bias repair fidelity and increase mutagenic pressure. IL-6/signal transducer and activator of transcription 3 (STAT3) pathways can modulate the expression of DNA repair enzymes and chromatin regulators, attenuating high-fidelity repair pathways. In particular, inflammatory STAT3 activation has been linked to repression of homologous recombination components, including breast cancer type 1 susceptibility protein (BRCA1) and RAD51 recombinase, alongside altered mismatch repair activity and a bias towards error-prone end-joining [58]. In parallel, STAT3 and NF-κB recruit histone acetyltransferases and chromatin-modifying enzymes to inflammatory gene loci, driving widespread chromatin remodelling and increased transcriptional accessibility [59]. Type I interferons further complicate this interplay. Interferon signalling can induce apolipoprotein B mRNA editing enzyme catalytic polypeptide-like 3 (APOBEC3) enzymes; for example, viral nucleic-acid sensing triggers STAT2-dependent APOBEC3A expression downstream of RIG-I/MAVS/IRF3 signalling [60]. Furthermore, DNA instability and replication stress themselves can trigger APOBEC3B expression and activity via ATR/CHK1 signalling, coupling fork stress to APOBEC-driven mutagenesis [61], and APOBEC3 induction can itself exacerbate DNA replication stress and chromosomal instability [62]. In parallel, the interferon-stimulated factor SAM and HD domain-containing protein 1 (SAMHD1) shapes deoxynucleotide triphosphate (dNTP) homeostasis and genome stability. SAMHD1 deficiency causes dNTP imbalance, chronic DNA damage signalling, senescence and upregulation of interferon-stimulated genes [63], while SAMHD1 also acts at stalled replication forks to limit release of ssDNA and thereby prevent cGAS-STING-driven type I interferon induction [64]. Through these combined mechanisms, inflammatory signalling not only damages DNA chemically but can also reprogramme the DDR itself, locking cells into a persistent damage-response mode that sustains both genomic instability and inflammatory output.

The dialogue between DNA instability and inflammation extends beyond individual cells into a multicellular network of stress propagation. Damaged or senescent cells, through the SASP, release cytokines, chemokines, proteases and extracellular vesicles (EVs) that remodel their surroundings and propagate inflammation (figure 1(7)). These secretions act as paracrine amplifiers, recruiting immune cells and inducing secondary DNA damage in neighbouring cells through ROS, RNS and inflammatory signalling. Immune cells, in turn, perpetuate this cycle: macrophages and neutrophils recruited to senescent niches release reactive species and proteases that compromise tissue integrity and provoke further genotoxic stress. Our own work has highlighted macrophages as key amplifiers of this circuit. In mice carrying an ERCC1-XPF defect specifically in monocytes and tissue-infiltrating macrophages, persistent DNA damage triggers Golgi dispersal, ER dilation, autophagy and robust exosome biogenesis [65]. The resulting macrophage-derived EVs reprogramme glucose uptake and mitochondrial respiration in recipient cells, driving insulin-independent glucose transport and high oxygen consumption. This metabolic rewiring, while acutely adaptive, increases intracellular glucose and activates mechanistic target of rapamycin (mTOR)-dependent inflammatory responses, thereby coupling genotoxic stress in macrophages to systemic metabolic inflammation and tissue pathology. Our recent findings demonstrate that persistent DNA damage in macrophages can directly drive immune autoreactivity through aberrant nuclear antigen presentation. Genome instability promotes the misrouting of nuclear components into antigen-processing pathways, resulting in sustained activation of autoreactive T cells and chronic inflammation [66]. In the brain, microglia activated by neuronal DNA damage and cytosolic nucleic acids secrete cytokines and metabolites that exacerbate neuronal genome instability, forming a feedback loop that drives neuroinflammation and neurodegeneration, as discussed in our recent work on DNA instability and neurodegenerative disease [19]. Extracellular vesicles further extend this communication network. EVs derived from damaged or senescent cells carry DNA fragments, mitochondrial components and inflammatory RNAs that activate cGAS-STING or RIG-I-like receptors in recipient cells, even at distant sites. Circulating EV-associated DNA, including R-loop-derived ssDNAs and telomeric DNA fragments, is increasingly recognized as a mobile vector of the DDR, disseminating inflammatory and metabolic stress throughout the organism [30].

Inflammation-driven mitochondrial dysfunction can amplify genotoxicity through increased ROS production, impaired mitophagy and release of mitochondrial nucleic acids, thereby reinforcing innate sensing pathways and the persistence of sterile inflammation [67]. Mitochondrial dysfunction magnifies this systemic dialogue: cytokine-driven mitochondrial ROS production and mtDNA release engage inflammasomes such as absent in melanoma 2 (AIM2) and NOD-, LRR- and pyrin domain-containing protein 3 (NLRP3), reinforcing the perception of persistent injury [68–70]. Together, these processes reveal inflammation as both a consequence and a catalyst of genomic instability, while simultaneously enforcing transcriptional and metabolic states that perpetuate inflammatory output. Through ROS/RNS production, cytokine-driven reprogramming of the DDR, R-loop formation and the systemic spread of genotoxic signals via EVs, inflammation transforms transient immune defence into a chronic, organism-wide assault on genome integrity. This reciprocal relationship between immune activation and DNA instability constitutes a central engine of inflammaging, one that links molecular stress to the progressive loss of tissue function with age. This completes the bidirectional logic of the damage–inflammation axis: DNA damage triggers innate immune signalling, and inflammation feeds back to perpetuate genomic instability (figure 1).

## 4. The self-amplifying damage–inflammation feedback loop

At the organismal level, ageing is not simply the passive accumulation of random lesions but the emergence of a self-sustaining stress network in which genomic instability and inflammation reinforce each other through persistent signalling and intercellular communication [28,71]. This damage–inflammation loop can convert transient stress responses into chronic pathology. The loop can initiate when persistent or irreparable lesions sustain DDR signalling, leading to a stable senescence programme with a pro-inflammatory secretory phenotype (SASP) [25]. Importantly, this framework implies that ageing reflects not only increasing damage burden, but also impaired resolution of stress programmes once they are triggered [3]. A key mechanistic bridge from damage to inflammation is the appearance of self-DNA in the cytosol, including cytosolic chromatin fragments and genome-instability-derived micronuclei, which engages cGAS-STING to drive inflammatory gene expression and paracrine senescence [27,29,40]. In parallel, senescent cells export inflammatory signals not only as soluble cytokines/chemokines but also via EVs and extracellular particles that transmit senescence and can carry DNA-associated components capable of activating cGAS-STING in recipient cells, with such DNA-containing particles detectable in the circulation of aged mice [72,73]. The resulting chronic inflammation, initially adaptive for clearance, becomes maladaptive when tissue repair or immune resolution fails. Pro-inflammatory cytokine milieus and immune-derived reactive species can induce new DNA lesions and interfere with repair capacity, including nitric oxide-dependent DNA damage with repair inhibition reported in inflammatory settings and disease models [48–50]. Chronic inflammation therefore becomes both a source of damage and a stabilizer of persistent DDR signalling. Mitochondrial dysfunction can amplify this process by increasing ROS and enabling mtDNA-dependent activation of cGAS-STING, thereby maintaining a continuing source of immunostimulatory self-DNA [28,74].

Comparative genetics offers a way to separate initiation from amplification within this loop. DNA repair disorders such as Cockayne syndrome and ERCC1-XPF-related progeroid conditions primarily impair lesion removal and transcription recovery, and their phenotypes support a model in which persistent DNA instability can be sufficient to initiate systemic ageing-like pathology. By contrast, interferonopathies such as Aicardi–Goutières syndrome, caused by defects in nucleic-acid metabolism or innate immune tolerance, demonstrate that chronic sensing of endogenous nucleic acids can generate inflammatory pathology even when the initiating lesion is immune recognition rather than a classical repair defect. These comparisons suggest that DNA damage and inflammatory sensing can enter the same circuit from different points. Agents that induce transcription-blocking damage, replication stress or mitochondrial injury tend to accelerate systemic decline, whereas interventions that reduce damage load or improve stress resolution, including dietary restriction in DNA repair-deficient mice, can delay progeroid pathology [9,64].

Thus, the nucleus, mitochondria and immune system engage in a circular ‘danger dialogue’ in which damage signalling and inflammation become tightly coupled phases of the same loop. At the cellular level, these feedbacks can sustain a chronic, self-reinforcing senescence/inflammatory state and propagate stress to neighbouring cells through cGAS-STING-driven SASP and EV-mediated paracrine signalling [27,72]. At the tissue level, this paracrine spread can convert localized injury into broader functional decline. Consistent with a causal contribution of senescent cells to age-related pathology, genetic clearance of p16Ink4a-positive senescent cells *in vivo* delays multiple ageing-associated disorders [75]. Over time, these reinforcing circuits manifest as inflammaging, systemic, low-grade inflammation associated with ageing [71], which can perpetuate tissue stress and predispose to age-related disease. Breaking this cycle, by improving genome maintenance, enhancing inflammatory resolution or dampening aberrant sensing of self-nucleic acids (notably cGAS-STING), emerges as a unifying therapeutic principle supported by genetic and pharmacologic evidence in ageing models [28,75].

## 5. Organismal consequences: from local lesions to systemic ageing

Although initiated at the cellular level, the damage–inflammation axis exerts its most profound effects at tissue and organismal scales (figure 2). With age, local instances of persistent DNA damage and immune activation become less effectively contained and can propagate via circulating mediators, EVs and nucleic-acid-associated signals, thereby transforming focal lesions into a body-wide network of chronic inflammation [76,77]. In addition to canonical EVs, senescent cells can release extracellular particles enriched in chromatin and DNA damage markers, providing a further route by which damage-associated signals can disseminate systemically and reinforce sterile inflammation [73]. A central implication of this systemic spread is that inflammaging is not merely the sum of independent local inflammatory sites, but an emergent organism-wide state sustained by mobile ‘stress vectors’. Circulating cell-free mitochondrial DNA (ccf-mtDNA) is increasingly recognized as a measurable systemic correlate of inflammation in older adults, with levels correlating with inflammatory markers such as IL-6, CRP and TNF signalling components [78].

**Figure 2.**
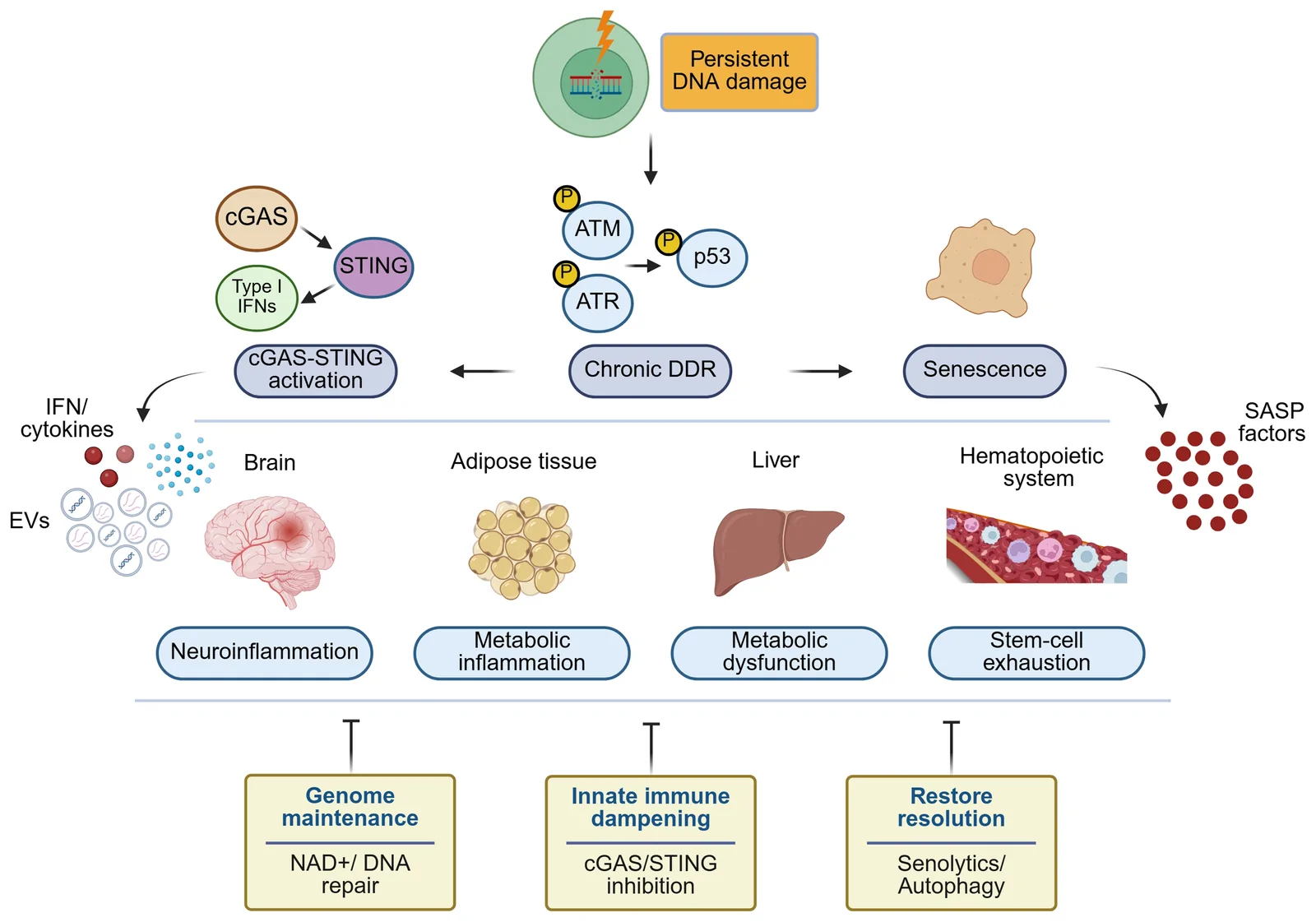
Systemic consequences of persistent DNA damage and therapeutic strategies to disrupt the damage–inflammation axis. Persistent DNA damage sustains chronic DNA damage response (DDR) signalling mediated by ATM, ATR and p53. Prolonged DDR promotes cellular senescence and activation of innate immune pathways, including cGAS-STING signalling and type I interferon production. Senescent cells secrete senescence-associated secretory phenotype (SASP) factors, while inflammatory cytokines and extracellular vesicles disseminate damage-associated signals beyond the initiating cell. These signals drive tissue-specific inflammatory and degenerative outcomes. In the brain, they promote neuroinflammation. In adipose tissue and liver, they induce metabolic inflammation and dysfunction. In the hematopoietic system, they contribute to stem-cell exhaustion. Together, these processes reinforce genome instability and chronic inflammation at the organismal level, accelerating ageing. Therapeutic strategies can target distinct nodes of this circuit by enhancing genome maintenance, dampening aberrant innate immune sensing or restoring inflammatory resolution through senolytic and autophagy-based interventions. (Created in BioRender: Theodorakis E. (2026) https://BioRender.com/wfd8lou.)

At the tissue level, persistent DDR and inflammatory signalling drive stem and progenitor cell dysfunction. In the hematopoietic system, sustained p53 activity constrains stem-cell state and function [79], while type I interferons drive hematopoietic stem cell (HSC) activation and can promote exhaustion when chronically engaged [80,81]. Chronic inflammatory cytokine exposure can further bias haematopoiesis towards myeloid differentiation at the expense of self-renewal [82]. In addition, an aged or senescent immune system can drive senescence and ageing-like dysfunction in solid organs, demonstrating that immune-cell intrinsic damage programmes can act systemically and in trans [8]. In epithelia such as intestine and skin, senescent niches and their SASP disrupt regenerative programmes and tissue organization. SASP factors from senescent stromal cells impair intestinal stem-cell differentiation and crypt formation [83], and sustained SASP exposure can reprogramme epithelial states *in vivo*, with prolonged signalling imposing growth arrest and dysfunction [84]. These processes are self-reinforcing, because inflammatory mediators can directly induce DNA instability and suppress repair capacity in neighbouring cells [49]. Moreover, systemic dissemination of SASP components is increasingly linked to age-associated organ dysfunction, supporting the view that the SASP acts not only locally but also as an endocrine-like signalling module during ageing [85].

The brain provides a striking example of tissue-level amplification. DNA instability can generate cytosolic DNA species that engage cGAS-STING signalling in microglia, promoting chemokine production and inflammatory migration programmes [86]. In disease-relevant contexts, microglial STING activation contributes to neuroinflammation and neurodegeneration [87], and pharmacologic inhibition of STING can alleviate microglial dysfunction and multiple Alzheimer's-like pathologies *in vivo* [88]. Importantly, EVs are increasingly implicated as mediators of brain–periphery inflammatory communication in ageing, and EVs secreted from senescent or stressed cells can induce inflammation and telomere dysfunction in recipient cells, reinforcing non-cell-autonomous genome instability [89].

In metabolic organs such as liver and adipose tissue, the damage–inflammation axis disrupts energy homeostasis. Persistent DNA damage in adipose tissue can trigger an ATM-dependent, chronic autoinflammatory programme that recruits immune cells and drives tissue degeneration [18]. In obesity models, mtDNA leakage can activate cGAS-STING signalling to promote adipose inflammation and insulin resistance [90]. In the liver, STING-IRF3 signalling can promote hepatocyte inflammation/apoptosis and metabolic dysfunction in non-alcoholic fatty liver disease (NAFLD) models [91]. Beyond nucleic acids, mitochondrial dysfunction also triggers systemic protein-based stress signals (‘mitokines’) that shape organismal inflammation. Growth differentiation factor 15 (GDF15), a mitochondrial stress-induced cytokine that rises with age, has been implicated as a key regulator of inflammatory and metabolic adaptation in ageing, linking mitochondrial stress to organismal resilience and systemic immune tone [92]. Mitochondrial dysfunction amplifies these processes: immunogenic mtDNA can act as an inflammatory trigger (e.g. via NLRP3 under appropriate contexts) [69], and circulating mtDNA levels rise with age and can stimulate pro-inflammatory outputs consistent with systemic ‘inflammaging’ [93]. Circulating nucleic acids and vesicle/particle-borne nucleic acids add a layer of systemic connectivity. Age-dependent changes in circulating EV pools (abundance/cargo/function) [76] and increased circulating mtDNA [93] provide mobile damage signals capable of engaging innate immune pathways in distant organs, contributing to a distributed, low-level alarm state across tissues.

Together, these findings indicate that the damage–inflammation axis spreads across organs through multiple interconnected carriers—including cytokines, EVs, circulating nucleic acids such as ccf-mtDNA and stress-induced endocrine signals such as GDF15. This distributed signalling architecture helps explain how local genome instability becomes organism-wide inflammaging, predisposing to multi-system decline. These systemic propagation routes and tissue-specific outcomes are summarized in figure 2.

## 6. The damage–inflammation axis in age-related diseases

The entanglement of DNA instability and inflammation manifests across diverse tissues, where local genomic instability and innate immune activation converge into organ-specific pathologies. Although the upstream circuitry often relies on shared sensing pathways, particularly detection of mislocalized nucleic acids and activation of interferon and NF-κB programmes, the downstream consequences are strongly shaped by tissue-specific architecture, metabolic demand and immune composition. This context dependence helps explain why similar damage–inflammation logic can produce neurodegeneration in the brain, metabolic dysfunction in peripheral organs and mutagenic microenvironments in proliferative epithelia [94].

In the brain, neuronal DNA instability and mitochondrial dysfunction can promote microglial activation through innate DNA-sensing pathways. Microglia engage cGAS-STING-linked inflammatory programmes in response to damage-derived cues and cytosolic DNA species [86]. In models relevant to neurodegeneration, crosstalk between DNA instability and cGAS-STING signalling has been shown to drive neuroinflammation and dopaminergic neurodegeneration in Parkinson's disease [95], and STING activity contributes to neuroinflammation/neurodegeneration in α-synucleinopathy [87]. In Alzheimer's disease models, pharmacologic blockade of STING can alleviate microglial dysfunction and multiple disease-associated pathologies [88].

In proliferative tissues, chronic inflammation establishes a mutagenic environment. Activated immune cells, particularly neutrophils, induce oxidative lesions and DNA strand breaks in epithelial cells [46]. Nitric oxide-dependent inflammatory signalling can directly induce DNA damage while inhibiting repair in epithelial malignancy-relevant contexts [49] and *in vivo* inflammatory disease models [48]. IL-22-driven inflammatory circuits further promote nitric oxide-dependent DNA damage and dysplasia in colitis-associated cancer models [50]. Nitrative DNA lesions, such as 8-nitroguanine, are detectable in inflammation-associated carcinogenesis and serve as biomarkers for sustained inflammatory genotoxic stress [51]. A complementary inflammatory axis in epithelial disease involves S100A8/A9 (calprotectin), a highly abundant inflammatory heterodimer elevated in multiple inflammatory disorders and robustly upregulated with ageing across mammalian tissues [96]. Notably, S100A8/A9 can intersect directly with DNA damage biology, as intracellular calprotectin has been shown to facilitate DNA damage responses and influence cell fate decisions in damage-stressed settings. These findings broaden the damage–inflammation framework beyond classical cytokines by implicating inflammatory damage-associated molecular pattern (DAMP)-like proteins as amplifiers of genome stress and tissue pathology [97].

In metabolic and cardiovascular systems, persistent DNA damage and innate immune activation intersect with nutrient and energy signalling. In adipose tissue, DNA instability can trigger chronic autoinflammatory programmes that recruit immune cells and drive tissue degeneration [18]. In obesity, extracellular DNA released from adipocytes stimulates adipose inflammation and insulin resistance, providing a mechanism by which damage-associated nucleic acids amplify metabolic inflammation [98]. In the liver, STING-IRF3 signalling promotes hepatocyte inflammation and metabolic dysfunction in NAFLD models [91]. In parallel, mitochondrial dysfunction amplifies these processes by sustaining ROS production and releasing oxidized mtDNA capable of activating innate inflammatory pathways, including inflammasomes [69]. Inflammasome activation also connects genome instability to tissue injury through inflammatory cell death programmes. Gasdermin-mediated pyroptosis, executed by pore-forming proteins such as gasdermin D (GSDMD), promotes inflammatory cytokine release (notably IL-1β and IL-18) and tissue damage [99]. Pyroptosis has been increasingly implicated in degenerative and age-associated diseases, linking innate immune activation to chronic tissue dysfunction [100]. Importantly, recent findings suggest that gasdermin pathways can intersect functionally with nucleic-acid sensing circuits, highlighting mechanistic coupling between inflammasome outputs and cGAS-STING-driven inflammation [101].

In the vasculature, oxidative stress activates DDR signalling in endothelial cells, and ATM-dependent signalling has been linked to endothelial dysfunction and premature senescence, thereby connecting DDR to vascular pathology-associated inflammation and tissue decline [102]. Across these pathological settings, a unifying mechanistic paradigm emerges: persistent nuclear and mitochondrial damage generates cytosolic or extracellular nucleic-acid signals and DAMPs that activate innate immune pathways, while inflammatory mediators and reactive species feedback to exacerbate DNA instability. This coupling converts local molecular lesions into progressive tissue dysfunction and multiorgan decline. Collectively, these organ-level outcomes illustrate how the damage–inflammation axis becomes disease-specific through tissue context, immune composition and stress propagation routes.

## 7. Therapeutic interventions targeting the damage–inflammation circuit

If ageing and age-related diseases arise in part from a self-sustaining loop between DNA instability and inflammation, then breaking this circuit becomes a logical strategy for disease prevention and functional rejuvenation. Emerging interventions can be broadly organized into three interdependent pillars: (i) enhancing genome maintenance, (ii) dampening aberrant nucleic-acid sensing and inflammatory signalling, and (iii) restoring homeostatic resolution through senescence control and epigenetic rejuvenation. A key implication of this framework is that durable benefit may require targeting both the initiation and the propagation of damage signals, rather than suppressing any single inflammatory cytokine in isolation.

Enhancing genome maintenance and repair fidelity targets the primary trigger of the loop. Interventions that restore NAD⁺ availability can improve the capacity of cells to sustain repair and stress responses, in part by relieving age-associated constraints on poly [ADP-ribose] polymerase 1 (PARP1)-dependent repair and DNA damage accumulation [103] and by improving nuclear-mitochondrial communication that deteriorates with declining NAD⁺ during ageing [104]. Because mitochondrial dysfunction is a major source of damage-associated inflammatory triggers, targeting mitochondrial quality control is equally crucial. Urolithin A induces mitophagy *in vivo* and improves mitochondrial function across model systems [105], and in humans it is safe and elicits a molecular signature consistent with improved mitochondrial and cellular health [106]. These findings support mitophagy promotion as a route to reduce chronic stress signalling and limit generation of immunostimulatory mtDNA. Recent findings also point to cell-cycle transcriptional control as an actionable bridge between persistent DDR signalling and inflammatory output. Loss of proper DREAM complex-mediated repression after DNA damage can permit inappropriate gene expression programmes and promote inflammatory signalling linked to cytosolic DNA sensing [107]. Therapeutically, reinforcing DREAM-like repression (or mimicking its transcriptional output) represents a strategy to couple quiescence enforcement with suppression of sterile inflammatory programmes when DDR activation becomes chronic.

Dampening aberrant DNA sensing and inflammatory signalling addresses the second node of the loop: maladaptive recognition of self-derived nucleic acids. Small-molecule inhibition of cGAS can reduce interferon expression in relevant immune-cell contexts [108]. Importantly, modulation of this axis can also be achieved by controlling extracellular propagation of innate immune agonists. The extracellular enzyme ectonucleotide pyrophosphatase/phosphodiesterase 1 (ENPP1) hydrolyses cGAMP and thereby limits STING activation in immune cells, effectively acting as a checkpoint that shapes paracrine and systemic spread of nucleic-acid-derived inflammatory signalling [109]. In cancer models, ENPP1 inhibition can potentiate antitumour immunity, including in combination with radiotherapy, illustrating how regulating extracellular cGAMP metabolism can amplify immune activation when therapeutically desired [110]. Conversely, in ageing contexts where chronic STING engagement is maladaptive, ENPP1-cGAMP control highlights a broader principle: intercellular nucleic-acid signalling can be tuned not only by intracellular sensors but also by extracellular enzymatic ‘gatekeepers’ that regulate the longevity and range of inflammatory cues [111]. Multiple STING inhibitors are also advancing, with *in vivo* evidence that pharmacologic STING inhibition can attenuate inflammatory tissue injury [112] and suppress neuroinflammation and neurotoxicity in disease-relevant neurodegeneration models [113]. Additional approaches target inflammasome activation: selective inhibition of NLRP3 with MCC950 reduces inflammatory atherosclerotic lesion development *in vivo* [114], supporting inflammasome suppression to reduce downstream cytokine amplification. Because cGAS-STING is essential for antimicrobial defence, the therapeutic aim in ageing is context-dependent modulation rather than complete suppression, with the goal of restoring appropriate sensing thresholds without compromising protective immunity.

Restoring resolution and rejuvenation, the capacity to terminate stress programmes and re-establish homeostasis, constitutes the third pillar. Senolytic strategies that eliminate senescent cells reduce systemic inflammatory burden and improve physical function and survival in aged mice [115]. Translationally, senescence-targeting approaches are rapidly expanding, with multiple senolytic and senomorphic agents already tested or under evaluation across clinical indications, highlighting increasing feasibility of targeting senescent cell burden as a therapeutic node [116]. In parallel, senomorphics that suppress deleterious SASP components (rather than killing senescent cells) are being explored as strategies to reduce inflammatory propagation while preserving beneficial aspects of acute senescence programmes [117]. Autophagy-enhancing interventions also intersect with this pillar. Rapamycin extends lifespan when initiated late in life [118] and is widely used experimentally to promote autophagy and dampen chronic inflammatory signalling. More broadly, autophagy and lysosomal quality control sit at a convergence point between genome stability and innate sensing, as they regulate clearance of damaged organelles, cytosolic DNA-containing structures and inflammatory signalling complexes. Finally, epigenetic rejuvenation approaches suggest that aspects of biological ageing are reversible. Partial reprogramming can ameliorate age-associated hallmarks *in vivo* [119], and targeted Oct4, Sox2 and Klf4 (OSK) expression can restore youthful epigenetic information and improve function in CNS tissue contexts such as vision restoration [120]. These results support the principle that ageing phenotypes driven by chronic stress signalling may be mitigated not only by reducing damage input, but also by resetting long-lived transcriptional and chromatin states that stabilize inflammation. Together, these strategies embody a shift in gerotherapeutics from targeting isolated hallmarks to disrupting the self-reinforcing circuitry that connects genome instability and inflammation. Enhancing genome integrity reduces triggers; dampening innate sensing prevents propagation; and restoring resolution rebuilds homeostasis, collectively defining a next generation of interventions acting at the nexus of DNA instability and inflammation. This circuit-level logic, and the main therapeutic intervention nodes, are summarized in figure 2.

## 8. Perspectives and future directions

The relationship between genome instability and inflammation reframes ageing as a persistent stress state driven by pathways that evolved for repair, defence and survival. The damage–inflammation axis encapsulates a central paradox of ageing biology: signalling pathways that promote survival through rapid repair and immune defence become chronic drivers of dysfunction when their resolution fails. In this view, ageing reflects the persistence of stress programmes beyond their adaptive window, transforming transient protective responses into long-term pathological states.

A major conceptual challenge is to disentangle causality within this circular relationship. Does persistent DNA damage initiate inflammaging, or does chronic inflammation erode genome stability? The most likely answer is context-dependent. DNA repair syndromes such as Cockayne syndrome argue that unrepaired transcription-blocking lesions can initiate systemic ageing-like changes, whereas interferonopathies such as Aicardi–Goutières syndrome indicate that chronic nucleic-acid sensing can drive pathology from the immune-recognition side of the circuit. Mouse models and interventions further support this bidirectionality: genotoxic treatments, defective lesion processing and mitochondrial stress can accelerate systemic decline, while dietary restriction and other stress-resolution strategies can delay accelerated ageing in DNA repair-deficient animals [9]. Importantly, this suggests that ageing may involve threshold-dependent transitions, whereby tissues cross from a resilient, repair-capable state into a self-amplifying inflammatory regime that is difficult to reverse. Identifying these transition points represents a key frontier for both mechanistic understanding and therapeutic intervention.

Disentangling these dynamics will require temporally resolved, multi-omic approaches capable of distinguishing cause from consequence *in vivo*. Emerging tools make this feasible. Single-cell and spatial transcriptomic technologies now allow mapping of DDR activation, innate immune signalling and senescence programmes within ageing tissues at unprecedented resolution. Proteomic profiling of EVs and circulating nucleic acids can reveal how genotoxic signals travel between organs. Integrating these data with AI-driven ageing clocks and systems-biology models may allow quantification of the damage–inflammation axis as a biomarker of biological age. Conceptually, the field must move beyond studying DNA instability and inflammation as separate hallmarks to address their coupled dynamics, how they co-evolve, amplify and stabilize over time.

Therapeutically, the goal shifts from repairing every lesion or suppressing every cytokine to restoring the capacity for resolution, reinstating the transient nature of repair and immune responses that defines youthful homeostasis. Strategies that enhance genome maintenance or suppress aberrant innate sensing may be most effective before the axis becomes fully self-sustaining, whereas later stages of ageing may require interventions that actively dismantle stabilized inflammatory circuits, such as senescence-targeting or epigenetic reprogramming approaches. This distinction suggests that gerotherapeutics should be evaluated not only by their molecular targets, but also by their capacity to shift tissues back across resolution thresholds towards homeostasis.

From an evolutionary perspective, signalling networks that ensure survival during reproduction, rapid DDR activation, inflammatory repair and immune vigilance may become maladaptive later in life. Ageing can thus be viewed as an evolutionary overshoot of survival pathways, in which failure to silence the DDR–immune interface becomes a molecular root of chronic disease. By viewing ageing as a persistent alarm state, genome instability, inflammation and degeneration emerge as interconnected facets of a single, self-reinforcing process. Understanding how this process is initiated, stabilized and potentially reversed will be critical for redefining ageing biology and for designing interventions that extend healthspan rather than merely suppressing individual symptoms.

## Data Availability

This article has no additional data.

## Theme

One contribution of 21 to a theme issue ‘Developmental circuits in ageing’.

## References

[1] Siametis A , Garinis GA. 2025 From genome to geroscience: how DNA damage shapes systemic decline. Bioessays 47, e700051. (doi:10.1002/bies.70051) PMC12455273 40785252

[2] Arvanitaki ES , Stratigi K, Garinis GA. 2022 DNA damage, inflammation and aging: insights from mice. Front. Aging 3, 973781. (doi:10.3389/fragi.2022.973781) PMC9490123 36160606

[3] Schumacher B , Pothof J, Vijg J, Hoeijmakers JHJ. 2021 The central role of DNA damage in the ageing process. Nature 592, 695–703. (doi:10.1038/s41586-021-03307-7) PMC9844150 33911272

[4] Stratigi K , Chatzidoukaki O, Garinis GA. 2017 DNA damage-induced inflammation and nuclear architecture. Mech. Ageing Dev. 165, 17–26. (doi:10.1016/j.mad.2016.09.008) 27702596

[5] Garinis GA , Schwer B, Schumacher B. 2017 Editorial: DNA damage & immunity. Mech. Ageing Dev. 165, 1–2. (doi:10.1016/j.mad.2017.04.006) PMC5544113 28445698

[6] Chatzinikolaou G , Karakasilioti I, Garinis GA. 2014 DNA damage and innate immunity: links and trade-offs. Trends Immunol. 35, 429–435. (doi:10.1016/j.it.2014.06.003) 25023467

[7] Rieckher M , Garinis GA, Schumacher B. 2021 Molecular pathology of rare progeroid diseases. Trends Mol. Med. 27, 907–922. (doi:10.1016/j.molmed.2021.06.011) 34272172

[8] Yousefzadeh MJ *et al.* 2021 An aged immune system drives senescence and ageing of solid organs. Nature 594, 100–105. (doi:10.1038/s41586-021-03547-7) PMC8684299 33981041

[9] Vermeij WP *et al.* 2016 Restricted diet delays accelerated ageing and genomic stress in DNA-repair-deficient mice. Nature 537, 427–431. (doi:10.1038/nature19329) PMC5161687 27556946

[10] Chatzidoukaki O , Goulielmaki E, Schumacher B, Garinis GA. 2020 DNA damage response and metabolic reprogramming in health and disease. Trends Genet. 36, 777–791. (doi:10.1016/j.tig.2020.06.018) 32684438

[11] Apostolou Z , Chatzinikolaou G, Stratigi K, Garinis GA. 2019 Nucleotide excision repair and transcription-associated genome instability. Bioessays 41, 1800201. (doi:10.1002/bies.201800201) 30919497

[12] Agathangelou K , Apostolou Z, Garinis GA. 2018 Nuclear DNA damage and ageing. Subcell. Biochem. 90, 309–322. (doi:10.1007/978-981-13-2835-0_10) 30779013

[13] Gyenis A *et al.* 2023 Genome-wide RNA polymerase stalling shapes the transcriptome during aging. Nat. Genet. 55, 268–279. (doi:10.1038/s41588-022-01279-6) PMC9925383 36658433

[14] Garinis GA , van der Horst GTJ, Vijg J, Hoeijmakers JHJ. 2008 DNA damage and ageing: new-age ideas for an age-old problem. Nat. Cell Biol. 10, 1241–1247. (doi:10.1038/ncb1108-1241) PMC4351702 18978832

[15] Garinis GA . 2008 Nucleotide excision repair deficiencies and the somatotropic axis in aging. Hormones 7, 9–16. (doi:10.14310/horm.2002.1111032) 18359739

[16] van der Pluijm I *et al.* 2007 Impaired genome maintenance suppresses the growth hormone–insulin-like growth factor 1 axis in mice with Cockayne syndrome. PLoS Biol. 5, e2. (doi:10.1371/journal.pbio.0050002) PMC1698505 17326724

[17] Niedernhofer LJ *et al.* 2006 A new progeroid syndrome reveals that genotoxic stress suppresses the somatotroph axis. Nature 444, 1038–1043. (doi:10.1038/nature05456) 17183314

[18] Karakasilioti I *et al.* 2013 DNA damage triggers a chronic autoinflammatory response, leading to fat depletion in NER progeria. Cell Metab. 18, 403–415. (doi:10.1016/j.cmet.2013.08.011) PMC4116679 24011075

[19] Arvanitaki ES *et al.* 2024 Microglia-derived extracellular vesicles trigger age-related neurodegeneration upon DNA damage. Proc. Natl Acad. Sci. USA 121, e2317402121. (doi:10.1073/pnas.2317402121) PMC11047102 38635632

[20] Ioannidou A , Goulielmaki E, Garinis GA. 2016 DNA damage: from chronic inflammation to age-related deterioration. Front. Genet. 7, 187. (doi:10.3389/fgene.2016.00187) PMC5078321 27826317

[21] Lans H , Hoeijmakers JHJ, Vermeulen W, Marteijn JA. 2019 The DNA damage response to transcription stress. Nat. Rev. Mol. Cell Biol. 20, 766–784. (doi:10.1038/s41580-019-0169-4) 31558824

[22] Blackford AN , Jackson SP. 2017 ATM, ATR, and DNA-PK: the trinity at the heart of the DNA damage response. Mol. Cell 66, 801–817. (doi:10.1016/j.molcel.2017.05.015) 28622525

[23] Tresini M *et al.* 2015 The core spliceosome as target and effector of non-canonical ATM signalling. Nature 523, 53–58. (doi:10.1038/nature14512) PMC4501432 26106861

[24] Coppé J-P , Desprez P-Y, Krtolica A, Campisi J. 2010 The senescence-associated secretory phenotype: the dark side of tumor suppression. Annu. Rev. Pathol. 5, 99–118. (doi:10.1146/annurev-pathol-121808-102144) PMC4166495 20078217

[25] Rodier F *et al.* 2009 Persistent DNA damage signalling triggers senescence-associated inflammatory cytokine secretion. Nat. Cell Biol. 11, 973–979. (doi:10.1038/ncb1909) PMC2743561 19597488

[26] Tilstra JS *et al.* 2012 NF-kappaB inhibition delays DNA damage-induced senescence and aging in mice. J. Clin. Invest. 122, 2601–2612. (doi:10.1172/JCI45785) PMC3386805 22706308

[27] Gluck S *et al.* 2017 Innate immune sensing of cytosolic chromatin fragments through cGAS promotes senescence. Nat. Cell Biol. 19, 1061–1070. (doi:10.1038/ncb3586) PMC5826565 28759028

[28] Gulen MF *et al.* 2023 cGAS-STING drives ageing-related inflammation and neurodegeneration. Nature 620, 374–380. (doi:10.1038/s41586-023-06373-1) PMC10412454 37532932

[29] Mackenzie KJ *et al.* 2017 cGAS surveillance of micronuclei links genome instability to innate immunity. Nature 548, 461–465. (doi:10.1038/nature23449) PMC5870830 28738408

[30] Siametis A , Stratigi K, Giamaki D, Chatzinikolaou G, Akalestou-Clocher A, Goulielmaki E, Luke B, Schumacher B, Garinis GA. 2024 Transcription stress at telomeres leads to cytosolic DNA release and paracrine senescence. Nat. Commun. 15, 4087. (doi:10.1038/s41467-024-48443-6) PMC11094137 38744897

[31] Chatzidoukaki O *et al.* 2021 R-loops trigger the release of cytoplasmic ssDNAs leading to chronic inflammation upon DNA damage. Sci. Adv. 7, eabj5769. (doi:10.1126/sciadv.abj5769) PMC8604417 34797720

[32] Kujirai T , Zierhut C, Takizawa Y, Kim R, Negishi L, Uruma N, Hirai S, Funabiki H, Kurumizaka H. 2020 Structural basis for the inhibition of cGAS by nucleosomes. Science 370, 455–458. (doi:10.1126/science.abd0237) PMC7584773 32912999

[33] Chiappinelli KB *et al.* 2015 Inhibiting DNA methylation causes an interferon response in cancer via dsRNA including endogenous retroviruses. Cell 162, 974–986. (doi:10.1016/j.cell.2015.07.011) PMC4556003 26317466

[34] Liddicoat BJ , Piskol R, Chalk AM, Ramaswami G, Higuchi M, Hartner JC, Li JB, Seeburg PH, Walkley CR. 2015 RNA editing by ADAR1 prevents MDA5 sensing of endogenous dsRNA as nonself. Science 349, 1115–1120. (doi:10.1126/science.aac7049) PMC5444807 26275108

[35] Crossley MP *et al.* 2024 Author correction: R-loop-derived cytoplasmic RNA-DNA hybrids activate an immune response. Nature 626, E6. (doi:10.1038/s41586-024-07064-1) 38263519

[36] Stetson DB , Ko JS, Heidmann T, Medzhitov R. 2008 Trex1 prevents cell-intrinsic initiation of autoimmunity. Cell 134, 587–598. (doi:10.1016/j.cell.2008.06.032) PMC2626626 18724932

[37] Crow YJ *et al.* 2006 Mutations in genes encoding ribonuclease H2 subunits cause Aicardi–Goutieres syndrome and mimic congenital viral brain infection. Nat. Genet. 38, 910–916. (doi:10.1038/ng1842) 16845400

[38] Li T , Chen ZJ. 2018 The cGAS-cGAMP-STING pathway connects DNA damage to inflammation, senescence, and cancer. J. Exp. Med. 215, 1287–1299. (doi:10.1084/jem.20180139) PMC5940270 29622565

[39] Hatch EM , Fischer AH, Deerinck TJ, Hetzer MW. 2013 Catastrophic nuclear envelope collapse in cancer cell micronuclei. Cell 154, 47–60. (doi:10.1016/j.cell.2013.06.007) PMC3749778 23827674

[40] Dou Z *et al.* 2017 Cytoplasmic chromatin triggers inflammation in senescence and cancer. Nature 550, 402–406. (doi:10.1038/nature24050) PMC5850938 28976970

[41] Liu Y , Zhang B, Duan R, Liu Y. 2024 Mitochondrial DNA leakage and cGas/STING pathway in microglia: crosstalk between neuroinflammation and neurodegeneration. Neuroscience 548, 1–8. (doi:10.1016/j.neuroscience.2024.04.009) 38685462

[42] Miller KN , Victorelli SG, Salmonowicz H, Dasgupta N, Liu T, Passos JF, Adams PD. 2021 Cytoplasmic DNA: sources, sensing, and role in aging and disease. Cell 184, 5506–5526. (doi:10.1016/j.cell.2021.09.034) PMC8627867 34715021

[43] Newman LE , Shadel GS. 2023 Mitochondrial DNA release in innate immune signaling. Annu. Rev. Biochem. 92, 299–332. (doi:10.1146/annurev-biochem-032620-104401) PMC11058562 37001140

[44] Lonkar P , Dedon PC. 2011 Reactive species and DNA damage in chronic inflammation: reconciling chemical mechanisms and biological fates. Int. J. Cancer 128, 1999–2009. (doi:10.1002/ijc.25815) PMC3334345 21387284

[45] Kay J , Thadhani E, Samson L, Engelward B. 2019 Inflammation-induced DNA damage, mutations and cancer. DNA Repair 83, 102673. (doi:10.1016/j.dnarep.2019.102673) PMC6801086 31387777

[46] Knaapen AM , Schins RP, Polat D, Becker A, Borm PJ. 2002 Mechanisms of neutrophil-induced DNA damage in respiratory tract epithelial cells. Mol. Cell. Biochem. 234, 143–151. (doi:10.1007/978-1-4615-1087-1_16) 12162427

[47] Butin-Israeli V *et al.* 2019 Neutrophil-induced genomic instability impedes resolution of inflammation and wound healing. J. Clin. Invest. 129, 712–726. (doi:10.1172/JCI122085) PMC6355304 30640176

[48] Ding X , Hiraku Y, Ma N, Kato T, Saito K, Nagahama M, Semba R, Kuribayashi K, Kawanishi S. 2005 Inducible nitric oxide synthase-dependent DNA damage in mouse model of inflammatory bowel disease. Cancer Sci. 96, 157–163. (doi:10.1111/j.1349-7006.2005.00024.x) PMC11160000 15771618

[49] Jaiswal M , LaRusso NF, Burgart LJ, Gores GJ. 2000 Inflammatory cytokines induce DNA damage and inhibit DNA repair in cholangiocarcinoma cells by a nitric oxide-dependent mechanism. Cancer Res. 60, 184–190. 10646872

[50] Wang C *et al.* 2017 Interleukin-22 drives nitric oxide-dependent DNA damage and dysplasia in a murine model of colitis-associated cancer. Mucosal. Immunol. 10, 1504–1517. (doi:10.1038/mi.2017.9) PMC5557711 28198364

[51] Hiraku Y , Kawanishi S. 2009 Immunohistochemical analysis of 8-nitroguanine, a nitrative DNA lesion, in relation to inflammation-associated carcinogenesis. Methods Mol. Biol. 574, 3–10. (doi:10.1007/978-1-60327-530-9_1) 19347269

[52] Acosta JC *et al.* 2013 A complex secretory program orchestrated by the inflammasome controls paracrine senescence. Nat. Cell Biol. 15, 978–990. (doi:10.1038/ncb2784) PMC3732483 23770676

[53] Ragu S , Matos-Rodrigues G, Lopez BS. 2020 Replication stress, DNA damage, inflammatory cytokines and innate immune response. Genes 11. 409. (doi:10.3390/genes11040409) PMC7230342 32283785

[54] Hartung M L , Gruber D C, Koch K N, Grüter L, Rehrauer H, Tegtmeyer N, Backert S, Müller A. 2015 *H. pylori*-induced DNA strand breaks are introduced by nucleotide excision repair endonucleases and promote NF-kappaB target gene expression. Cell Rep. 13, 70–79. (doi:10.1016/j.celrep.2015.08.074) 26411687

[55] Mangerich A *et al.* 2012 Infection-induced colitis in mice causes dynamic and tissue-specific changes in stress response and DNA damage leading to colon cancer. Proc. Natl Acad. Sci. USA 109, E1820–1829. (doi:10.1073/pnas.1207829109) PMC3390855 22689960

[56] Sahan AZ , Hazra TK, Das S. 2018 The pivotal role of DNA repair in infection mediated-inflammation and cancer. Front. Microbiol. 9, 663. (doi:10.3389/fmicb.2018.00663) PMC5904280 29696001

[57] Sohn JJ *et al.* 2012 Macrophages, nitric oxide and microRNAs are associated with DNA damage response pathway and senescence in inflammatory bowel disease. PLoS ONE 7, e44156. (doi:10.1371/journal.pone.0044156) PMC3435404 22970173

[58] Barbarulo A *et al.* 2013 Poly(ADP-ribose) polymerase family member 14 (PARP14) is a novel effector of the JNK2-dependent pro-survival signal in multiple myeloma. Oncogene 32, 4231–4242. (doi:10.1038/onc.2012.448) 23045269

[59] Calado DP *et al.* 2010 Constitutive canonical NF-kappaB activation cooperates with disruption of BLIMP1 in the pathogenesis of activated B cell-like diffuse large cell lymphoma. Cancer Cell 18, 580–589. (doi:10.1016/j.ccr.2010.11.024) PMC3018685 21156282

[60] Oh S *et al.* 2021 Genotoxic stress and viral infection induce transient expression of APOBEC3A and pro-inflammatory genes through two distinct pathways. Nat. Commun. 12, 4923. (doi:10.1038/s41467-021-25203-4) PMC8363607 34389714

[61] Kanu N *et al.* 2016 DNA replication stress mediates APOBEC3 family mutagenesis in breast cancer. Genome Biol. 17, 185. (doi:10.1186/s13059-016-1042-9) PMC5025597 27634334

[62] Venkatesan S *et al.* 2021 Induction of APOBEC3 exacerbates DNA replication stress and chromosomal instability in early breast and lung cancer evolution. Cancer Discov. 11, 2456–2473. (doi:10.1158/2159-8290.CD-20-0725) PMC8487921 33947663

[63] Kretschmer S *et al.* 2015 SAMHD1 prevents autoimmunity by maintaining genome stability. Ann. Rheum. Dis. 74, e17. (doi:10.1136/annrheumdis-2013-204845) PMC4345975 24445253

[64] Coquel F *et al.* 2018 SAMHD1 acts at stalled replication forks to prevent interferon induction. Nature 557, 57–61. (doi:10.1038/s41586-018-0050-1) 29670289

[65] Goulielmaki E *et al.* 2020 Tissue-infiltrating macrophages mediate an exosome-based metabolic reprogramming upon DNA damage. Nat. Commun. 11, 42. (doi:10.1038/s41467-019-13894-9) PMC6940362 31896748

[66] Niotis G *et al.* 2026 DNA damage in macrophages drives immune autoreactivity via nuclear antigen presentation. Nat. Aging 6, 393–413. (doi:10.1038/s43587-025-01053-3) PMC12920141 41540279

[67] Kawanishi S , Ohnishi S, Ma N, Hiraku Y, Murata M. 2017 Crosstalk between DNA damage and inflammation in the multiple steps of carcinogenesis. Int. J. Mol. Sci. 18, 1808. (doi:10.3390/ijms18081808) PMC5578195 28825631

[68] Zhou R , Yazdi AS, Menu P, Tschopp J. 2011 A role for mitochondria in NLRP3 inflammasome activation. Nature 469, 221–225. (doi:10.1038/nature09663) 21124315

[69] Shimada K *et al.* 2012 Oxidized mitochondrial DNA activates the NLRP3 inflammasome during apoptosis. Immunity 36, 401–414. (doi:10.1016/j.immuni.2012.01.009) PMC3312986 22342844

[70] Gu F *et al.* 2024 Microglial mitochondrial DNA release contributes to neuroinflammation after intracerebral hemorrhage through activating AIM2 inflammasome. Exp. Neurol. 382, 114950. (doi:10.1016/j.expneurol.2024.114950) 39278588

[71] Franceschi C , Bonafè M, Valensin S, Olivieri F, De Luca M, Ottaviani E, De Benedictis G. 2000 Inflamm-aging. An evolutionary perspective on immunosenescence. Ann. N. Y. Acad. Sci. 908, 244–254. (doi:10.1111/j.1749-6632.2000.tb06651.x) 10911963

[72] Borghesan M *et al.* 2019 Small extracellular vesicles are key regulators of non-cell autonomous intercellular communication in senescence via the interferon protein IFITM3. Cell Rep. 27, 3956–3971. (doi:10.1016/j.celrep.2019.05.095) PMC6613042 31242426

[73] Zaretski S , Nieto Torres JL, Lei X, Nolan JP, Hansen M, Adams PD. 2025 Senescent cells secrete chromatin components via senescence-associated extracellular particles. bioRxiv. (doi:10.64898/2025.12.12.694055)

[74] Rongvaux A *et al.* 2014 Apoptotic caspases prevent the induction of type I interferons by mitochondrial DNA. Cell 159, 1563–1577. (doi:10.1016/j.cell.2014.11.037) PMC4272443 25525875

[75] Baker DJ , Wijshake T, Tchkonia T, LeBrasseur NK, Childs BG, van de Sluis B, Kirkland JL, van Deursen JM. 2011 Clearance of p16Ink4a-positive senescent cells delays ageing-associated disorders. Nature 479, 232–236. (doi:10.1038/nature10600) PMC3468323 22048312

[76] Alibhai FJ *et al.* 2020 Cellular senescence contributes to age-dependent changes in circulating extracellular vesicle cargo and function. Aging Cell 19, e13103. (doi:10.1111/acel.13103) PMC7059145 31960578

[77] Xiao Y , Liang J, Witwer KW, Zhang Y, Wang Q, Yin H. 2022 Extracellular vesicle-associated microRNA-30b-5p activates macrophages through the SIRT1/NF-kappaB pathway in cell senescence. Front. Immunol. 13, 955175. (doi:10.3389/fimmu.2022.955175) PMC9471260 36119099

[78] Nidadavolu LS *et al.* 2023 Associations between circulating cell-free mitochondrial DNA, inflammatory markers, and cognitive and physical outcomes in community dwelling older adults. Immun. Ageing 20, 19. (doi:10.1186/s12979-023-00342-y) PMC10204157 37221566

[79] Liu Y *et al.* 2009 p53 regulates hematopoietic stem cell quiescence. Cell Stem Cell 4, 37–48. (doi:10.1016/j.stem.2008.11.006) PMC2839936 19128791

[80] Essers MA , Offner S, Blanco-Bose WE, Waibler Z, Kalinke U, Duchosal MA, Trumpp A. 2009 IFNalpha activates dormant haematopoietic stem cells* in vivo*. Nature 458, 904–908. (doi:10.1038/nature07815) 19212321

[81] Sato T , Onai N, Yoshihara H, Arai F, Suda T, Ohteki T. 2009 Interferon regulatory factor-2 protects quiescent hematopoietic stem cells from type I interferon-dependent exhaustion. Nat. Med. 15, 696–700. (doi:10.1038/nm.1973) 19483695

[82] Pietras EM *et al.* 2016 Chronic interleukin-1 exposure drives haematopoietic stem cells towards precocious myeloid differentiation at the expense of self-renewal. Nat. Cell Biol. 18, 607–618. (doi:10.1038/ncb3346) PMC4884136 27111842

[83] Yun J *et al.* 2023 Senescent cells perturb intestinal stem cell differentiation through Ptk7 induced noncanonical Wnt and YAP signaling. Nat. Commun. 14, 126. (doi:10.1038/s41467-022-35487-9) PMC9834240 36631445

[84] Ritschka B , Storer M, Mas A, Heinzmann F, Ortells MC, Morton JP, Sansom OJ, Zender L, Keyes WM. 2017 The senescence-associated secretory phenotype induces cellular plasticity and tissue regeneration. Genes Dev. 31, 172–183. (doi:10.1101/gad.290635.116) PMC5322731 28143833

[85] Sutanto H , Fetarayani D, Narendra MR, Nasution SA. 2025 The role of the senescence-associated secretory phenotype in cardiovascular disease among the elderly. Eur. J. Intern. Med. 141, 106488. (doi:10.1016/j.ejim.2025.106488) 40858450

[86] Talbot EJ , Joshi L, Thornton P, Dezfouli M, Tsafou K, Perkinton M, Khoronenkova SV. 2024 cGAS-STING signalling regulates microglial chemotaxis in genome instability. Nucleic Acids Res. 52, 1188–1206. (doi:10.1093/nar/gkad1184) PMC10853792 38084916

[87] Hinkle JT *et al.* 2022 STING mediates neurodegeneration and neuroinflammation in nigrostriatal alpha-synucleinopathy. Proc. Natl Acad. Sci. USA 119, e2118819119. (doi:10.1073/pnas.2118819119) PMC9169780 35394877

[88] Chung S , Jeong JH, Park JC, Han JW, Lee Y, Kim JI, Mook-Jung I. 2024 Blockade of STING activation alleviates microglial dysfunction and a broad spectrum of Alzheimer's disease pathologies. Exp. Mol. Med. 56, 1936–1951. (doi:10.1038/s12276-024-01295-y) PMC11447230 39218977

[89] Lananna BV , Imai SI. 2021 Friends and foes: extracellular vesicles in aging and rejuvenation. FASEB Bioadv. 3, 787–801. (doi:10.1096/fba.2021-00077) PMC8493967 34632314

[90] Bai J *et al.* 2017 DsbA-L prevents obesity-induced inflammation and insulin resistance by suppressing the mtDNA release-activated cGAS-cGAMP-STING pathway. Proc. Natl Acad. Sci. USA 114, 12196–12201. (doi:10.1073/pnas.1708744114) PMC5699051 29087318

[91] Qiao JT *et al.* 2018 Activation of the STING-IRF3 pathway promotes hepatocyte inflammation, apoptosis and induces metabolic disorders in nonalcoholic fatty liver disease. Metabolism 81, 13–24. (doi:10.1016/j.metabol.2017.09.010) 29106945

[92] Moon JS *et al.* 2020 Growth differentiation factor 15 protects against the aging-mediated systemic inflammatory response in humans and mice. Aging Cell 19, e13195. (doi:10.1111/acel.13195) PMC7431835 32691494

[93] Pinti M *et al.* 2014 Circulating mitochondrial DNA increases with age and is a familiar trait: implications for ‘inflamm-aging’. Eur. J. Immunol. 44, 1552–1562. (doi:10.1002/eji.201343921) 24470107

[94] Li X , Li C, Zhang W, Wang Y, Qian P, Huang H. 2023 Inflammation and aging: signaling pathways and intervention therapies. Signal Transduct. Target Ther. 8, 239. (doi:10.1038/s41392-023-01502-8) PMC10248351 37291105

[95] Khan S , Delotterie DF, Xiao J, Thangavel R, Hori R, Koprich J, Alway SE, McDonald MP, Khan MM. 2025 Crosstalk between DNA damage and cGAS-STING immune pathway drives neuroinflammation and dopaminergic neurodegeneration in Parkinson's disease. Brain Behav. Immun. 130, 106065. (doi:10.1016/j.bbi.2025.106065) PMC12412893 40752659

[96] Swindell WR , Johnston A, Xing X, Little A, Robichaud P, Voorhees JJ, Fisher G, Gudjonsson JE. 2013 Robust shifts in S100a9 expression with aging: a novel mechanism for chronic inflammation. Sci. Rep. 3, 1215. (doi:10.1038/srep01215) PMC3564041 23386971

[97] Argyris PP , Saavedra F, Malz C, Stone IA, Wei Y, Boyle WS, Johnstone KF, Khammanivong A, Herzberg MC. 2023 Intracellular calprotectin (S100A8/A9) facilitates DNA damage responses and promotes apoptosis in head and neck squamous cell carcinoma. Oral Oncol. 137, 106304. (doi:10.1016/j.oraloncology.2022.106304) PMC9877195 36608459

[98] Nishimoto S *et al.* 2016 Obesity-induced DNA released from adipocytes stimulates chronic adipose tissue inflammation and insulin resistance. Sci. Adv. 2, e1501332. (doi:10.1126/sciadv.1501332) PMC4820373 27051864

[99] Burdette BE , Esparza AN, Zhu H, Wang S. 2021 Gasdermin D in pyroptosis. Acta Pharm. Sin. B 11, 2768–2782. (doi:10.1016/j.apsb.2021.02.006) PMC8463274 34589396

[100] Zhou J , Qiu J, Song Y, Liang T, Liu S, Ren C, Song X, Cui L, Sun Y. 2023 Pyroptosis and degenerative diseases of the elderly. Cell Death. Dis. 14, 94. (doi:10.1038/s41419-023-05634-1) PMC9908978 36755014

[101] Cheng CK , Yi M, Wang L, Huang Y. 2024 Role of gasdermin D in inflammatory diseases: from mechanism to therapeutics. Front. Immunol. 15, 1456244. (doi:10.3389/fimmu.2024.1456244) PMC11381298 39253076

[102] Zhan H , Suzuki T, Aizawa K, Miyagawa K, Nagai R. 2010 Ataxia telangiectasia mutated (ATM)-mediated DNA damage response in oxidative stress-induced vascular endothelial cell senescence. J. Biol. Chem. 285, 29662–29670. (doi:10.1074/jbc.M110.125138) PMC2937997 20639198

[103] Li J *et al.* 2017 A conserved NAD(+) binding pocket that regulates protein-protein interactions during aging. Science 355, 1312–1317. (doi:10.1126/science.aad8242) PMC5456119 28336669

[104] Gomes AP *et al.* 2013 Declining NAD(+) induces a pseudohypoxic state disrupting nuclear-mitochondrial communication during aging. Cell 155, 1624–1638. (doi:10.1016/j.cell.2013.11.037) PMC4076149 24360282

[105] Ryu D *et al.* 2016 Urolithin A induces mitophagy and prolongs lifespan in *C. elegans* and increases muscle function in rodents. Nat. Med. 22, 879–888. (doi:10.1038/nm.4132) 27400265

[106] Andreux PA , Blanco-Bose W, Ryu D, Burdet F, Ibberson M, Aebischer P, Auwerx J, Singh A, Rinsch C. 2019 The mitophagy activator urolithin A is safe and induces a molecular signature of improved mitochondrial and cellular health in humans. Nat. Metab. 1, 595–603. (doi:10.1038/s42255-019-0073-4) 32694802

[107] Feng H , Lane KA, Roumeliotis TI, Jeggo PA, Somaiah N, Choudhary JS, Downs JA. 2022 PBAF loss leads to DNA damage-induced inflammatory signaling through defective G2/M checkpoint maintenance. Genes Dev. 36, 790–806. (doi:10.1101/gad.349249.121) PMC9480851 35902118

[108] Wiser C , Kim B, Vincent J, Ascano M. 2020 Small molecule inhibition of human cGAS reduces total cGAMP output and cytokine expression in cells. Sci. Rep. 10, 7434. (doi:10.1038/s41598-020-64348-y) PMC7200739 32371942

[109] Huang R , Ning Q, Zhao J, Zhao X, Zeng L, Yi Y, Tang S. 2024 Targeting ENPP1 for cancer immunotherapy: killing two birds with one stone. Biochem. Pharmacol. 220, 116006. (doi:10.1016/j.bcp.2023.116006) 38142838

[110] Baird JR *et al.* 2024 A novel small molecule Enpp1 inhibitor improves tumor control following radiation therapy by targeting stromal Enpp1 expression. Sci. Rep. 14, 29634. (doi:10.1038/s41598-024-80677-8) PMC11612208 39622844

[111] Wang SN *et al.* 2025 ENPP1 inhibitor with ultralong drug-target residence time as an innate immune checkpoint blockade cancer therapy. Cell Rep. Med. 6, 102336. (doi:10.1016/j.xcrm.2025.102336) PMC12490234 40914167

[112] Kobritz M , Borjas T, Patel V, Coppa G, Aziz M, Wang P. 2022 H151, a small molecule inhibitor of STING as a novel therapeutic in intestinal ischemia-reperfusion injury. Shock 58, 241–250. (doi:10.1097/SHK.0000000000001968) PMC9489661 35959789

[113] Wang B , Wang Y, Qiu J, Gao S, Yu S, Sun D, Lou H. 2023 The STING inhibitor C-176 attenuates MPTP-induced neuroinflammation and neurodegeneration in mouse Parkinsonian models. Int. Immunopharmacol. 124, 110827. (doi:10.1016/j.intimp.2023.110827) 37619411

[114] van der Heijden T , Kritikou E, Venema W, van Duijn J, van Santbrink PJ, Slutter B, Foks AC, Bot I, Kuiper J. 2017 NLRP3 inflammasome inhibition by MCC950 reduces atherosclerotic lesion development in apolipoprotein E-deficient mice—brief report. Arterioscler. Thromb. Vasc. Biol. 37, 1457–1461. (doi:10.1161/ATVBAHA.117.309575) 28596375

[115] Xu M *et al.* 2018 Senolytics improve physical function and increase lifespan in old age. Nat. Med. 24, 1246–1256. (doi:10.1038/s41591-018-0092-9) PMC6082705 29988130

[116] Suda M , Paul KH, Tripathi U, Minamino T, Tchkonia T, Kirkland JL. 2024 Targeting cell senescence and senolytics: novel interventions for age-related endocrine dysfunction. Endocr. Rev. 45, 655–675. (doi:10.1210/endrev/bnae010) PMC11405506 38500373

[117] Saliev T , Singh PB. 2025 Targeting senescence: a review of senolytics and senomorphics in anti-aging interventions. Biomolecules 15, 860. (doi:10.3390/biom15060860) PMC12190739 40563501

[118] Harrison DE *et al.* 2009 Rapamycin fed late in life extends lifespan in genetically heterogeneous mice. Nature 460, 392–395. (doi:10.1038/nature08221) PMC2786175 19587680

[119] Ocampo A *et al.* 2016 *In vivo* amelioration of age-associated hallmarks by partial reprogramming. Cell 167, 1719–1733. (doi:10.1016/j.cell.2016.11.052) PMC5679279 27984723

[120] Lu Y *et al.* 2020 Reprogramming to recover youthful epigenetic information and restore vision. Nature 588, 124–129. (doi:10.1038/s41586-020-2975-4) PMC7752134 33268865

